# The global burden of tuberculous meningitis in adults: A modelling study

**DOI:** 10.1371/journal.pgph.0000069

**Published:** 2021-12-08

**Authors:** Peter J. Dodd, Muhammad Osman, Fiona V. Cresswell, Anna M. Stadelman, Nguyen Huu Lan, Nguyen Thuy Thuong Thuong, Morris Muzyamba, Lisa Glaser, Sicelo S. Dlamini, James A. Seddon

**Affiliations:** 1 School of Health and Related Research, University of Sheffield, Sheffield, United Kingdom; 2 Desmond Tutu TB Centre, Department of Paediatrics and Child Health, Stellenbosch University, Stellenbosch, South Africa; 3 London School of Hygiene and Tropical Medicine, London, United Kingdom; 4 Infectious Diseases Institute, Kampala, Uganda; 5 MRC-UVRI-LSHTM Uganda Research Unit, Entebbe, Uganda; 6 School of Public Health, University of Minnesota, Minneapolis, MN, United States of America; 7 Pham Ngoc Thach Hospital, Ho Chi Minh City, Vietnam; 8 Oxford University Clinical Research Unit, Ho Chi Minh City, Vietnam; 9 Nuffield Department of Medicine, University of Oxford, Oxford, United Kingdom; 10 Tuberculosis Section, National Infection Service, Public Health England, London, United Kingdom; 11 Research Information Monitoring, Evaluation, and Surveillance, National Tuberculosis Control and Management Cluster, National Department of Health, Pretoria, South Africa; 12 Department of Infectious Diseases, Imperial College London, London, United Kingdom; Pontificia Universidad Católica de Chile: Pontificia Universidad Catolica de Chile, CHILE

## Abstract

Tuberculous meningitis (TBM) is the most lethal form of tuberculosis. The incidence and mortality of TBM is unknown due to diagnostic challenges and limited disaggregated reporting of treated TBM by existing surveillance systems. We aimed to estimate the incidence and mortality of TBM in adults (15+ years) globally. Using national surveillance data from Brazil, South Africa, the United Kingdom, the United States of America, and Vietnam, we estimated the fraction of reported tuberculosis that is TBM, and the case fatality ratios for treated TBM in each of these countries. We adjusted these estimates according to findings from a systematic review and meta-analysis and applied them to World Health Organization tuberculosis notifications and estimates to model the global TBM incidence and mortality. Assuming the case detection ratio (CDR) for TBM was the same as all TB, we estimated that in 2019, 164,000 (95% UI; 129,000–199,000) adults developed TBM globally; 23% were among people living with HIV. Almost 60% of incident TBM occurred in males and 20% were in adults 25–34 years old. 70% of global TBM incidence occurred in Southeast Asia and Africa. We estimated that 78,200 (95% UI; 52,300–104,000) adults died of TBM in 2019, representing 48% of incident TBM. TBM case fatality in those treated was on average 27%. Sensitivity analysis assuming improved detection of TBM compared to other forms of TB (CDR odds ratio of 2) reduced estimated global mortality to 54,900 (95% UI; 32,200–77,700); assuming instead worse detection for TBM (CDR odds ratio of 0.5) increased estimated mortality to 125,000 (95% UI; 88,800–161,000). Our results highlight the need for improved routine TBM monitoring, especially in high burden countries. Reducing TBM incidence and mortality will be necessary to achieve the End TB Strategy targets.

## Introduction

Following inhalation of aerosolized *M*. *tuberculosis*, most individuals either eradicate or contain the organism through innate and adaptive immune mechanisms [1]. Failure of these mechanisms, however, can result in haematogenous dissemination of bacilli to distant sites, including to the meninges surrounding the brain [1–4]. Once bacilli replicate within the meninges and cause local inflammation, the clinical condition of tuberculous meningitis (TBM) results. In the wake of the HIV epidemic, in high tuberculosis prevalence countries *M*. *tuberculosis* is now the second leading cause of meningitis after Cryptococcus [5, 6]. TBM is universally fatal if untreated and even when treated is associated with high mortality [7–9]. Amongst survivors, severe permanent disability is common with a substantial cost to individuals, families, and society [7, 8]. In addition, the time from symptom onset to death is much more rapid than for other forms of tuberculosis.

TBM can cause a diverse clinical picture including altered mental status, meningitic features, seizures, cranial nerve palsies, and focal neurological deficits [10–12]. Many of these features overlap with other conditions [10], and consequently TBM may not be readily considered if a patient seeks care. Diagnosing TBM comes with several additional challenges, which are most acute in low- and middle-income countries (LMICs). First, diagnosis can only be made using cerebrospinal fluid (CSF), requiring a lumbar puncture [13, 14]. Secondly, once a sample is obtained, processing requires appropriate laboratory capacity. Finally, even with the best laboratory infrastructure, the currently available diagnostic tests (smear microscopy, molecular tests including Xpert MTB/RIF, or CSF culture) are only moderately sensitive and commonly yield false negative results [15]. These diagnostic obstacles, combined with the rapid progression to death if untreated mean that many individuals with TBM die undiagnosed [16]. Evidence from a high tuberculosis/HIV context found 15% (1348/8759) of meningitis cases had CSF white cells >20/μL with lymphocytic predominance and negative diagnostic work-up, much of which is likely undiagnosed TBM [17].

Our understanding of the burden of TBM is limited. In addition to the burden of undiagnosed TBM, data on those diagnosed with TBM are not routinely captured. Currently, health programmes report tuberculosis patients as having any pulmonary involvement or being exclusively extrapulmonary [18], meaning a patient with TBM and pulmonary tuberculosis is classified as pulmonary tuberculosis. The World Health Organization (WHO) employs modelling approaches to estimate the overall burden of incident tuberculosis and tuberculosis deaths using methods that incorporate notifications, case detection estimates, prevalence surveys, estimates of case fatality ratios, and vital registration [19]. TBM is not specifically included in these approaches and a better understanding of TBM burden may influence overall estimates of global tuberculosis mortality given the high mortality associated with TBM. The Global Burden of Diseases study by the Institute for Health Metrics and Evaluation (IHME) has estimated the global burden of meningitis and the relative contribution of different causative organisms, but did not include TBM [20].

Due to underdiagnosis and paucity of population-level data on treated TBM there is substantial uncertainty about the true incidence, mortality, and societal impact of the disease. A better understanding the burden of TBM could help the WHO, Ministries of Health, National Tuberculosis Programmes, and other partner organisations prioritize resources and identify training needs, and researchers plan future studies. Highlighting the discrepancy between expected and diagnosed TBM is also an important step in programmatic evaluation, to advocate for programmatic support.

The natural history and clinical manifestations of tuberculosis are different in children and in adults [21], with young children having a higher risk of progression to disease following infection and a higher risk of developing severe, disseminated forms of disease [22]. The presentation of TBM in young children is less charactersitic than in adults, and the diagnosis is less commonly confirmed [23]. In addition, Bacillus Calmette–Guérin (BCG) vaccination has some efficacy against TBM in young children [16], an effect not confirmed in adults. These factors lead to key epidemiological differences between children and adults in terms of tuberculosis and TBM, therefore we chose to focus on adults. In this study we model the global incidence and mortality of adult TBM in different WHO regions, across a range of ages, and by sex and HIV status.

## Methods

### Fraction of tuberculosis that is TBM and case fatality ratios

To estimate the proportion of tuberculosis that is TBM (defined by ICD-10 code A17.0), and their case fatality ratio (CFR) on treatment, we sought national surveillance data that included counts by year, age, and sex, stratified by HIV status for: notified tuberculosis, notified TBM, and deaths among notified TBM patients. Data did not always include all categories. We obtained data for Brazil (19 years, 777,611 tuberculosis patients, 9,267 TBM patients, TBM deaths unavailable), South Africa (8 years, 2,155,572 tuberculosis patients, 32,362 TBM patients, 5,067 TBM deaths), United Kingdom (20 years, 139,450 tuberculosis patients, 2,840 TBM patients, TBM deaths unavailable), United States of America (5 years, 44,176 tuberculosis patients, 676 TBM patients, 183 TBM deaths), and Vietnam (5 years, 62,487 tuberculosis patients, 3,447 TBM patients, 601 TBM deaths). Most countries used 10-year age categories from 15 years up to 65+ years. Brazil used different age categories and did not stratify by sex. United Kingdom data were all from England. Vietnam comprised two data sets: one by year but without TBM deaths, and another without year not including tuberculosis notifications (numbers above sum over both datasets). Further information is shown in Table 1.

**Table 1:** Number of people included in meta-analysis by characteristic (rows) and type (columns). Percentages of counts within strata are shown in brackets. Blanks indicate no data.

Stratum	Value	Tuberculosis	TBM	TBM deaths
Total	Total	3179296 (100%)	48592 (100%)	5851 (100%)
Sex	missing	777611 (24%)	9267 (19%)	
	male	1344697 (42%)	19258 (40%)	3160 (54%)
	female	1056988 (33%)	20067 (41%)	2691 (46%)
HIV	HIV-	1609560 (51%)	14046 (29%)	855 (15%)
	HIV+	1569736 (49%)	34546 (71%)	4996 (85%)
Country	BRA	777611 (24%)	9267 (19%)	
	GBR	139450 (4%)	2840 (6%)	
	USA	44176 (1%)	676 (1%)	183 (3%)
	VNM	62487 (2%)	1786 (4%)	
	VNM (deaths)		1661 (3%)	601 (10%)
	ZAF	2155572 (68%)	32362 (67%)	5067 (87%)
Age (BRA)	15–19	47236 (6%)	305 (3%)	
	20–39	381408 (49%)	4668 (50%)	
	40–59	257834 (33%)	3511 (38%)	
	60–64	33832 (4%)	328 (4%)	
	65–69	22960 (3%)	225 (2%)	
	70–79	25506 (3%)	188 (2%)	
	80+	8835 (1%)	42 (0%)	
Age (not BRA)	15–24	318274 (13%)	4767 (12%)	443 (8%)
	25–34	734746 (31%)	13378 (34%)	1915 (33%)
	35–44	645725 (27%)	11556 (29%)	1794 (31%)
	45–54	388890 (16%)	5731 (15%)	979 (17%)
	55–64	191213 (8%)	2448 (6%)	461 (8%)
	65+	122837 (5%)	1445 (4%)	259 (4%)
Year	2000	6291 (<1%)	69 (<1%)	
	2001	24676 (1%)	415 (1%)	
	2002	28109 (1%)	483 (1%)	
	2003	30352 (1%)	496 (1%)	
	2004	32058 (1%)	479 (1%)	
	2005	35761 (1%)	564 (1%)	
	2006	37189 (1%)	584 (1%)	
	2007	40511 (1%)	657 (1%)	
	2008	44464 (1%)	669 (1%)	
	2009	247192 (8%)	3953 (8%)	514 (9%)
	2010	317857 (10%)	5107 (11%)	774 (13%)
	2011	357356 (11%)	5464 (11%)	860 (15%)
	2012	344327 (11%)	5229 (11%)	772 (13%)
	2013	344021 (11%)	5121 (11%)	686 (12%)
	2014	363412 (11%)	5303 (11%)	664 (11%)
	2015	353144 (11%)	4392 (9%)	411 (7%)
	2016	320413 (10%)	4626 (10%)	501 (9%)
	2017	84950 (3%)	1193 (2%)	35 (1%)
	2018	88307 (3%)	1118 (2%)	33 (1%)
	2019	78906 (2%)	1009 (2%)	
	missing		1661 (3%)	601 (10%)

TBM: tuberculous meningitis; BRA: Brazil; GBR: Great Britain (data all from England); USA: United States of America; VNM: Vietnam; ZAF: South Africa

We used a two-step meta-analysis approach: first conducting country-level meta-analysis across years (to account for unmeasured random variation between years); second, to pool country-level estimates. For each country, we used random effects generalized linear meta-regression (with logit link and Poisson distribution) to estimate the proportion of tuberculosis incidence that is TBM and the TBM CFRs by age and sex (assuming no sex-age interaction). Only countries with relevant data were included in each step-two analysis. For Brazil, a restricted cubic spline meta-regression was used, fitted to the age category mid-points. Predictions and 95% prediction intervals were computed at the midpoints of the common age categories, and, lacking sex-stratification, these estimates were treated as pertaining to women. The country-level meta-analytic estimates were then meta-analysed for fractions by age and sex to produce pooled predictions and I^2^ used to quantify heterogeneity. For deaths in people living with HIV (PLHIV), only two countries had data, so an inverse variance-weighted mean was used for pooling. In South Africa, CFR in PLWHIV was lower than a previous systematic review and meta-analysis estimates by Stadelman and colleagues [7]. We scaled up the South African estimates of CFR in PLWHIV so that the weighted average CFR matched the previously reported value.

### Estimates of TBM incidence and mortality

**Fig 1:**
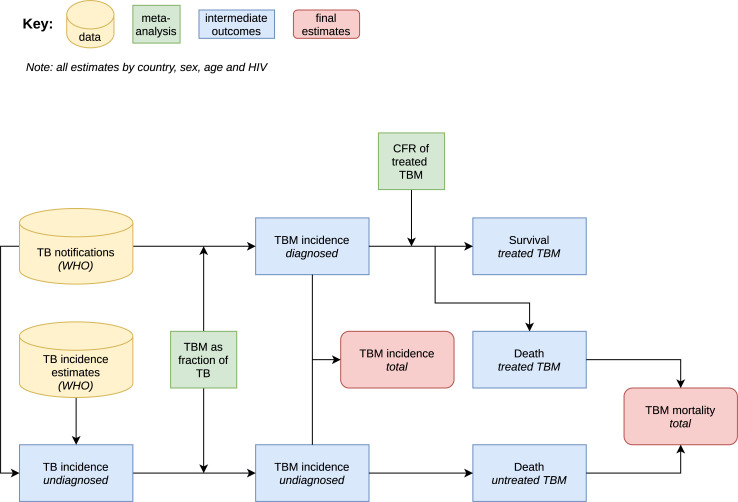
Flow chart of overall methods. TB: tuberculosis; TBM: tuberculous meningitis; CRF: Case fatality Rate; WHO: World Health Organization.

To estimate TBM incidence and mortality in people aged 15 years and over for each country in 2019, stratified by age, sex, and HIV status, we followed the procedure shown in the schematic in Fig 1. We used WHO-collated tuberculosis notification data stratified by age and sex, and WHO estimates of tuberculosis incidence in the corresponding sex and age group. We assumed that notified tuberculosis patients received treatment, and that the gap between incidence and notification represented patients who did not receive effective treatment. We used the WHO estimates of HIV prevalence in incident tuberculosis to stratify tuberculosis incidence by HIV status and assumed this applied equally to both treated and untreated tuberculosis. We then applied our meta-analytic estimates of the fraction of incident tuberculosis that is TBM by sex and age-group to both treated and untreated incident tuberculosis, therefore assuming TBM has the same likelihood of treatment as other forms of tuberculosis. As a sensitivity analysis, we considered higher and lower odds of detection and treatment for TBM compared to all tuberculosis, using odds ratios of 2 and 0.5. Finally, we applied our meta-analytic CFRs for treated TBM to the portion of incidence that received treatment and assumed that untreated TBM always results in death.

Uncertainty was included for each of the inputs (tuberculosis incidence estimates; proportion of tuberculosis with HIV; meta-analytic proportion of tuberculosis that is TBM; and CFRs) by analytically propagating variances through calculations to calculate uncertainty intervals (UI) as 95% quantiles under a normal approximation. We report TBM incidence and mortality, stratified by age, sex, WHO region, HIV, and tuberculosis treatment status in the main article, and provide country-level estimates in S1 Appendix. We completed a GATHER checklist. All analysis was performed in R version 4.0.1; source code and input data are available at https://github.com/petedodd/tbme. The study was approved by the Stellenbosch University Health Research Ethics Committee (X20/11/042).

### Role of the funding source

The funders of the study had no role in study design, data analysis, data interpretation, or writing of the report.

## Results

### Fraction of tuberculosis that is TBM and case fatality ratios

**Fig 2:**
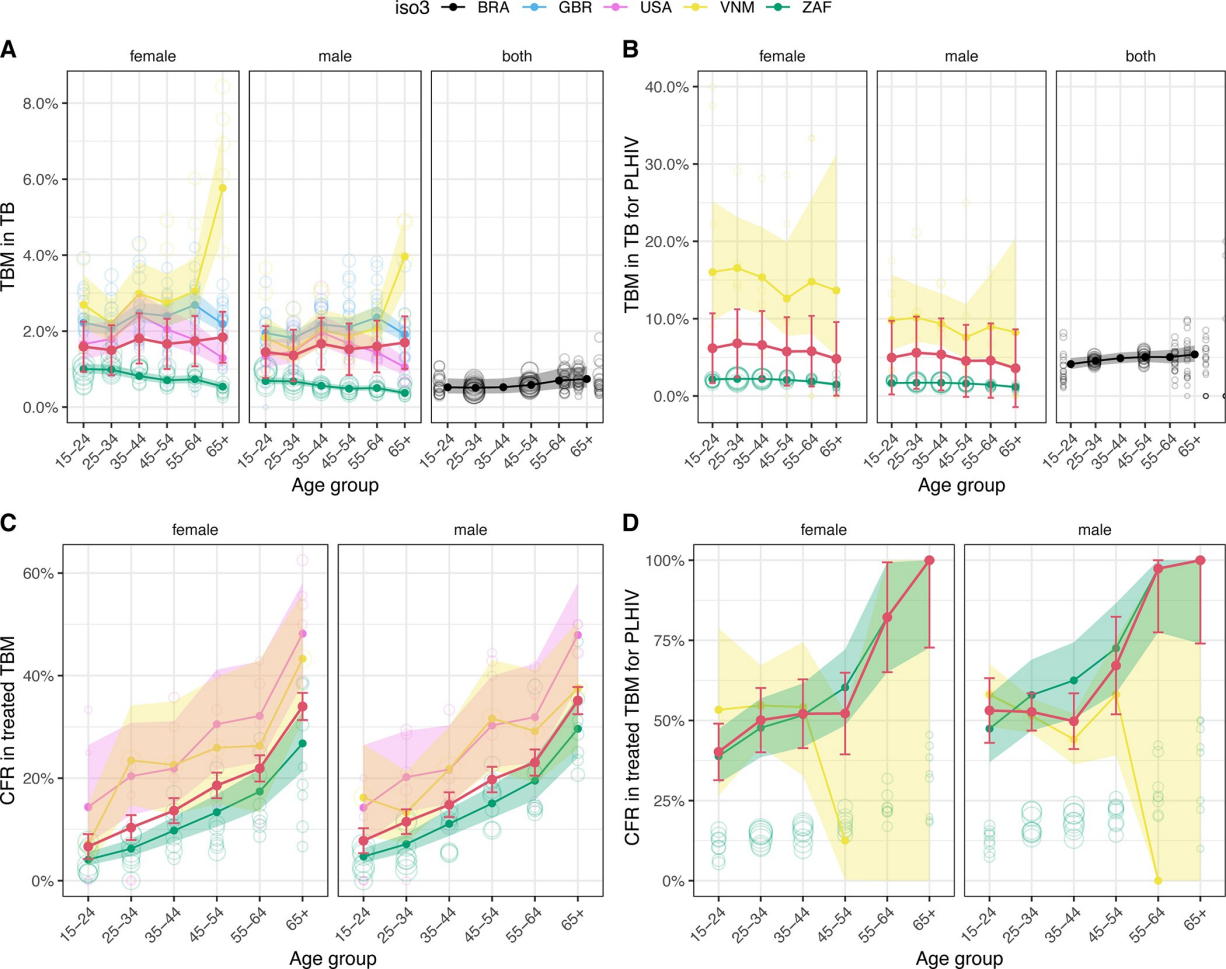
Age, sex, and HIV-stratified fraction of notified tuberculosis that is TB meningitis (top row, A & B), and case fatality ratio among treated TB meningitis (bottom row, C & D). HIV-negative (first column, A & C); HIV-positive (second column, B & D). Meta-analytic results for each country are shown by solid points and lines (central estimate) and shaded ribbons (95% confidence intervals). Open points show data for individual years in each country, with size proportional to denominator count. Pooled meta-analytic estimates are shown as red points, lines and 95% confidence interval error bars. (ISO3 country codes: ZAF = South Africa, GBR = United Kingdom, USA = United States of America, BRA = Brazil). Brazil has different age categories and no sex disaggregation. Map data from https://datacatalog.worldbank.org/search/dataset/0038272.

Across all ages and sexes, we estimated between 1.3% and 1.8% for pooled estimates of the proportion of tuberculosis that is TBM in HIV-negative people (Fig 2A; Table 1 in S1 Appendix), and between 3.6% and 6.8% in PLHIV (Fig 2B; Table 1 in S1 Appendix). Variation between countries was substantial (country-weighted mean range 0.5% to 2.3%; I^2^ = 98.6%). Women consistently showed higher proportions of incident tuberculosis that was TBM, but there were no clear patterns by age. CFRs increased steeply with age (Fig 2C and 2D) but did not show clear patterns by sex. The weighted average CFR for HIV-negative individuals was 15.9% (95% confidence interval [CI]; 13.4% - 18.3%), consistent with the review estimate of 16% (95%CI; 10% - 24%) from Stadelman and colleagues [7]. As noted in the methods, the weighted CFR for HIV-positive individuals was scaled to match the corresponding review estimate in Stadelman and colleagues, i.e. 57% (95%CI; 48% - 67%).

### Estimates of TBM incidence and mortality

**Fig 3:**
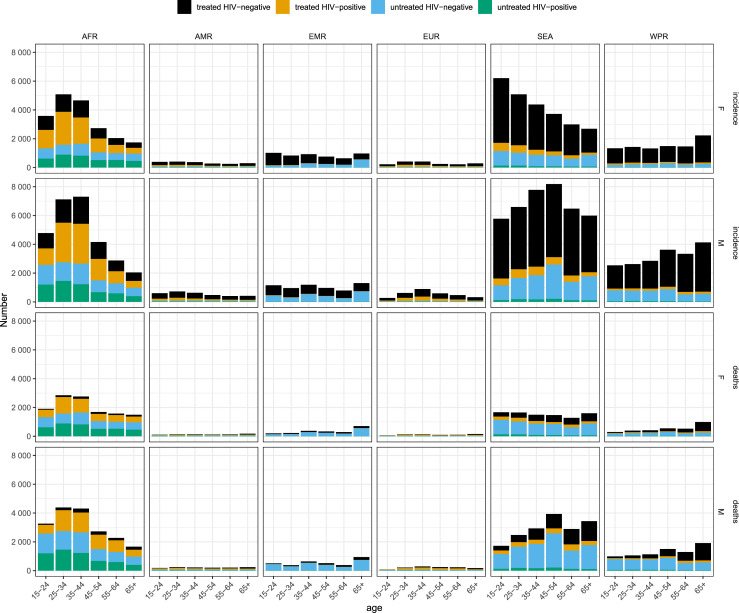
TBM incidence and deaths sex, age, WHO region, HIV-infection and anti-tuberculosis treatment status. TBM: tuberculous meningitis; AFR: African Region; AMR: Americas Region; EMR: Eastern Mediterranean Region; EUR: European Region: SEA: South East Asian Region: WPR: Western Pacific Region; WHO: World Health Organization; F: female; M: male.

We estimate that 164,000 (95% UI; 129,000–199,000) adults developed TBM in 2019, of whom 118,000 (95% UI; 85,400–150,000) or 72% were diagnosed and treated (Table 2). Of incident TBM, 63,200 (95% UI; 35,300–91,000) or 39% were women and 37,800 (95% UI; 19,700–55,900) or 23% were living with HIV (Table 2). The largest incidence was in the 25-34- and 35-44-year age groups: 31,900 (95% UI; 18,800–45,000) and 32,700 (95% UI; 19,600–45,900), respectively. However, all age groups had substantial incidence, including an incidence of 22,400 (95% UI; 12,000–32,900) among those over 65 years (Fig 3). The WHO region with the greatest burden of incident TBM was the Southeast Asia region with incidence of 65,900 (95% UI; 34,600–97,100) followed by the African region with incidence of 48,100 (95% UI; 41,400–54,800).

**Table 2:** Incidence and mortality of tuberculous meningitis, stratified by WHO region, HIV status and whether the patient received TBM treatment.

			WHO Region						
Metric	Treatment	HIV	AFR	AMR	EMR	EUR	SEA	WPR	Global
Incidence	Treated	Negative	12,000 (11,500–12,500)	3,190 (3,010–3,370)	7,110 (6,490–7,740)	3,030 (2,870–3,200)	45,000 (41,100–49,000)	21,200 (19,300–23,000)	91,600 (87,100–96,000)
		Positive	16,800 (11,900–21,700)	1,180 (0–2,570)	209 (0–4,550)	1,380 (0–2,790)	5,020 (0–33,300)	1,470 (0–14,600)	26,000 (0–58,000)
		Total	28,800 (23,900–33,700)	4,370 (2,970–5,760)	7,320 (2,930–11,700)	4,420 (3,000–5,830)	50,100 (21,500–78,700)	22,600 (9,420–35,900)	118,000 (85,400–150,000)
	Untreated	Negative	9,940 (7,240–12,600)	666 (375–958)	4,080 (1,470–6,680)	436 (68–804)	14,300 (1,780–26,900)	5,310 (1,130–9,500)	34,800 (21,000–48,500)
		Positive	9,370 (5,720–13,000)	241 (126–355)	112 (48–175)	149 (0–453)	1,470 (0–3,020)	410 (141–680)	11,800 (7,760–15,700)
		Total	19,300 (14,800–23,900)	907 (594–1,220)	4,190 (1,580–6,800)	585 (107–1,060)	15,800 (3,150–28,500)	5,720 (1,530–9,910)	46,500 (32,200–60,900)
	Total	Negative	22,000 (19,200–24,700)	3,850 (3,510–4,200)	11,200 (8,510–13,900)	3,470 (3,060–3,870)	59,400 (46,200–72,600)	26,500 (21,900–31,000)	126,000 (112,000–141,000)
		Positive	26,200 (20,100–32,300)	1,420 (28–2,810)	321 (0–4,660)	1,530 (95–2,970)	6,490 (0–34,800)	1,880 (0–15,000)	37,800 (5,610–70,000)
		Total	48,100 (41,400–54,800)	5,270 (3,840–6,710)	11,500 (6,410–16,600)	5,000 (3,510–6,500)	65,900 (34,600–97,100)	28,400 (14,500–42,200)	164,000 (129,000–199,000)
Mortality	Treated	Negative	1,880 (1,800–1,960)	543 (512–575)	1,190 (1,080–1,300)	545 (516–575)	7,680 (6,970–8,380)	4,350 (3,880–4,810)	16,200 (15,300–17,000)
		Positive	9,700 (6,890–12,500)	720 (0–1,590)	127 (0–2,920)	846 (0–1,700)	3,090 (0–21,300)	997 (0–11,800)	15,500 (0–37,100)
		Total	11,600 (8,770–14,400)	1,260 (391–2,140)	1,320 (0–4,110)	1,390 (533–2,250)	10,800 (0–29,000)	5,340 (0–16,100)	31,700 (10,100–53,300)
	Untreated	Negative	9,940 (7,240–12,600)	666 (375–958)	4,080 (1,470–6,680)	436 (68–804)	14,300 (1,780–26,900)	5,310 (1,130–9,500)	34,800 (21,000–48,500)
		Positive	9,370 (5,720–13,000)	241 (126–355)	112 (48–175)	149 (0–453)	1,470 (0–3,020)	410 (141–680)	11,800 (7,760–15,700)
		Total	19,300 (14,800–23,900)	907 (594–1,220)	4,190 (1,580–6,800)	585 (107–1,060)	15,800 (3,150–28,500)	5,720 (1,530–9,910)	46,500 (32,200–60,900)
	Total	Negative	11,800 (9,110–14,500)	1,210 (917–1,500)	5,270 (2,660–7,880)	982 (612–1,350)	22,000 (9,430–34,600)	9,660 (5,450–13,900)	51,000 (37,200–64,700)
		Positive	19,100 (14,500–23,700)	961 (81–1,840)	239 (0–3,030)	994 (84–1,900)	4,560 (0–22,900)	1,410 (0–12,200)	27,200 (5,280–49,200)
		Total	30,900 (25,600–36,200)	2,170 (1,240–3,100)	5,510 (1,690–9,320)	1,980 (993–2,960)	26,600 (4,350–48,800)	11,100 (0–22,600)	78,200 (52,300–104,000)

TBM: tuberculous meningitis; AFR: African Region; AMR: Americas Region; EMR: Eastern Mediterranean Region; EUR: European Region: SEA: Southeast Asian Region: WPR: Western Pacific Region; WHO: World Health Organization

**Fig 4:**
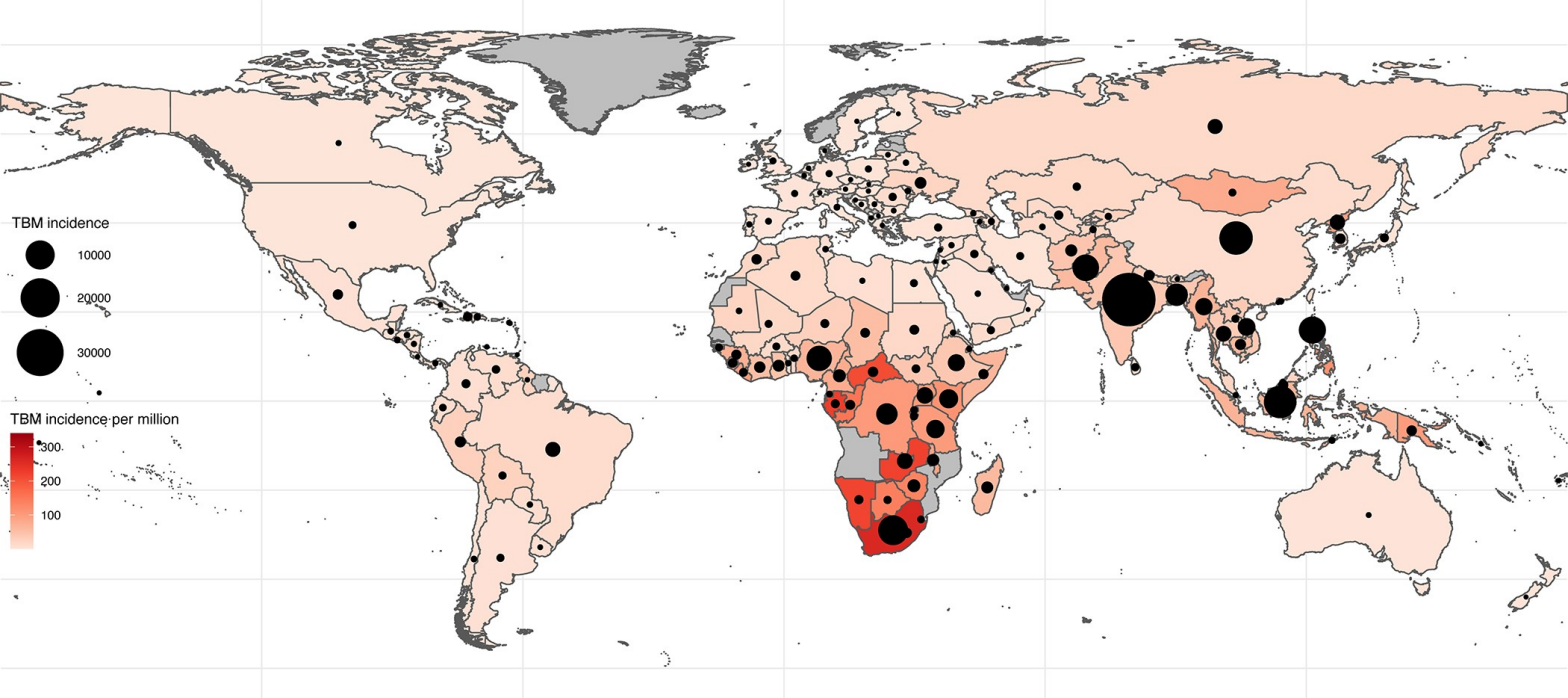
Absolute (dot size) and per capita incidence (colour) of TBM by country.

Our analysis suggests that 78,200 (95% UI; 52,300–104,000) adults died of TBM in 2019 (Table 1). Of the 118,000 individuals with incident TBM that were diagnosed and treated, 31,700 (95% UI; 10,100–53,300) died representing an overall CFR of 27%. All of the 46,500 (95% UI; 32,200–60,900) people with incident TBM that went undiagnosed, were assumed to die representing 59% of the overall TBM deaths. We estimate that 27,200 (95% UI; 5,280–49,200) adults with HIV died from TBM representing 35% of all TBM deaths, and we estimate that of all deaths, 36% (28,300, 95% UI; 18,400–38,300) were women (Table 2). The age group with the most deaths was the 35–45-year group with 14,900 (95% UI; 9,920–19,900) (Fig 3 and Table 3). The African and Southeast Asian WHO regions had the most deaths with 30,900 (95% UI; 25,600–36,200) and 26,600 (95% UI; 4,350–48,800) deaths, respectively. Country level estimates for incidence and mortality are provided in associated repository, and shown for incidence in Fig 4.

**Table 3:** Incidence and mortality of tuberculous meningitis, stratified by age band, HIV status and sex.

			Age bands (years)						
Metric	Sex	HIV	15–24	25–34	35–44	45–54	55–64	65+	Total
Incidence	Female	Negative	9,840 (2,740–16,900)	8,970 (3,660–14,300)	8,610 (4,270–13,000)	7,100 (3,600–10,600)	5,990 (2,990–8,990)	6,880 (3,590–10,200)	47,400 (20,800–73,900)
		Positive	2,920 (1,470–4,380)	4,290 (1,930–6,640)	3,480 (1,680–5,280)	2,120 (1,120–3,120)	1,630 (927–2,330)	1,340 (769–1,900)	15,800 (7,890–23,700)
		Total	12,800 (5,520–20,000)	13,300 (7,450–19,100)	12,100 (7,390–16,800)	9,220 (5,580–12,900)	7,620 (4,540–10,700)	8,220 (4,880–11,600)	63,200 (35,300–91,000)
	Male	Negative	11,800 (4,730–18,800)	12,900 (6,100–19,700)	15,200 (7,160–23,200)	14,600 (6,480–22,700)	11,900 (4,850–19,000)	12,600 (5,530–19,700)	79,000 (34,900–123,000)
		Positive	3,370 (1,990–4,750)	5,730 (3,020–8,430)	5,470 (2,700–8,240)	3,440 (1,810–5,060)	2,440 (1,370–3,510)	1,590 (951–2,230)	22,000 (11,800–32,200)
		Total	15,100 (7,960–22,300)	18,600 (11,300–26,000)	20,600 (12,200–29,100)	18,000 (9,750–26,300)	14,400 (7,210–21,500)	14,200 (7,100–21,300)	101,000 (55,500–146,000)
	Total	Negative	21,600 (7,470–35,700)	21,900 (9,760–34,000)	23,800 (11,400–36,100)	21,700 (10,100–33,300)	17,900 (7,840–28,000)	19,500 (9,120–29,900)	126,000 (55,700–197,000)
		Positive	6,290 (3,460–9,120)	10,000 (4,960–15,100)	8,940 (4,370–13,500)	5,550 (2,920–8,180)	4,070 (2,300–5,840)	2,930 (1,720–4,140)	37,800 (19,700–55,900)
		Total	27,900 (13,500–42,300)	31,900 (18,800–45,000)	32,700 (19,600–45,900)	27,200 (15,300–39,100)	22,000 (11,700–32,200)	22,400 (12,000–32,900)	164,000 (90,800–237,000)
Mortality	Female	Negative	2,610 (784–4,440)	2,730 (1,260–4,200)	3,070 (1,710–4,430)	2,850 (1,660–4,040)	2,460 (1,470–3,440)	3,780 (2,060–5,500)	17,500 (8,940–26,000)
		Positive	1,660 (937–2,380)	2,690 (1,410–3,970)	2,280 (1,240–3,310)	1,420 (809–2,020)	1,450 (829–2,070)	1,340 (769–1,900)	10,800 (5,990–15,700)
		Total	4,270 (2,300–6,230)	5,420 (3,470–7,370)	5,350 (3,640–7,060)	4,260 (2,930–5,600)	3,910 (2,740–5,070)	5,120 (3,310–6,930)	28,300 (18,400–38,300)
	Male	Negative	4,300 (1,880–6,720)	4,900 (2,460–7,350)	6,060 (3,200–8,920)	6,500 (2,950–10,100)	4,900 (2,550–7,250)	6,780 (3,440–10,100)	33,500 (16,500–50,400)
		Positive	2,460 (1,470–3,440)	3,850 (2,210–5,500)	3,480 (1,920–5,040)	2,630 (1,470–3,790)	2,400 (1,350–3,440)	1,590 (951–2,230)	16,400 (9,370–23,400)
		Total	6,760 (4,150–9,370)	8,760 (5,810–11,700)	9,540 (6,280–12,800)	9,140 (5,400–12,900)	7,290 (4,720–9,870)	8,370 (4,980–11,800)	49,900 (31,300–68,400)
	Total	Negative	6,910 (2,670–11,200)	7,630 (3,720–11,500)	9,130 (4,910–13,400)	9,350 (4,610–14,100)	7,350 (4,020–10,700)	10,600 (5,510–15,600)	51,000 (25,400–76,500)
		Positive	4,110 (2,400–5,820)	6,540 (3,620–9,470)	5,750 (3,160–8,350)	4,050 (2,280–5,810)	3,850 (2,180–5,520)	2,930 (1,720–4,140)	27,200 (15,400–39,100)
		Total	11,000 (6,450–15,600)	14,200 (9,280–19,100)	14,900 (9,920–19,900)	13,400 (8,330–18,500)	11,200 (7,460–14,900)	13,500 (8,280–18,700)	78,200 (49,700–107,000)

In the primary analysis we assumed that the case detection ratio (CDR) in TBM (the proportion of all estimated TBM that was diagnosed) was the same as the CDR in all tuberculosis (i.e. the detection odds ratio was 1). Our sensitivity analysis with an odds ratio of 2 (better detection for TBM) reduced global TBM incidence by 14% to 141,000 (95% UI; 108,000–174,000) and global TBM mortality by 30% to 54,900 (95% UI; 32,200–77,700) deaths (see Table 2 in S1 Appendix). Our analysis with worse detection for TBM (odds ratio of 0.5 compared to all tuberculosis) increased global TBM incidence by 29% to 211,000 (95% UI; 168,000–254,000) and global TBM mortality by 60% to 125,000 (95% UI; 88,800–161,000) deaths (see Table 3 in S1 Appendix).

## Discussion

This analysis demonstrated that around 1.5% of HIV-negative tuberculosis patients have TBM, and that this figure is higher for HIV-positive adults with tuberculosis at around 5%. The CFR increased with age for both HIV-positive and HIV-negative individuals and was similar for men and women. We estimated that 164,000 adults developed TBM worldwide in 2019, of whom 78,200 died. We found that 23% of the incidence and 35% of deaths were in HIV-positive individuals and that 72% of all incident TBM are diagnosed and treated each year. We estimate that 59% of adults that died of TBM were undiagnosed. The burden of incidence and deaths was highest in the younger age groups yet older individuals with treated TBM had higher CFRs than those younger so contributed relatively more deaths. Compared to women, men had a higher burden of incidence and deaths for all age groups and WHO regions, regardless of HIV status.

A previous review article estimated that the global TBM incidence in adults and children was 100,000/year [4]. The authors justified this estimate through two approaches. First, they combined the proportion of TBM in a large German cohort of tuberculosis patients (0.9%) [24] with the global estimate of tuberculosis incidence (10 million/year). Secondly, they used a Brazilian study which found that 6% of extrapulmonary tuberculosis was TBM [25], and then applied this first to the proportion of tuberculosis reported to the WHO globally that is extrapulmonary (15%) and then to the total WHO estimate of tuberculosis incidence. In the USA, TBM constituted 1.0% of all incident tuberculosis between 1993 and 2006 [26], however in Texas this proportion was up to 2.7% [27]. Extrapolating these data directly to tuberculosis and HIV endemic settings as well as LMICs is problematic as the TBM epidemiology is likely to be different. Our estimates of TBM incidence and mortality provide insight into the relative contribution of age, sex, HIV status, and geographic location. In addition, they indicate what proportion of TBM is diagnosed and treated. Given that our estimates do not include children, who are a high-risk group for TBM, we believe that the overall burden of TBM is substantially higher than the 100,000/year previously estimated.

The implications of our estimates need careful consideration. TBM is the most severe form of tuberculosis and contributes to substantial mortality. WHO estimates that 1.4 million individuals die of tuberculosis each year and our mortality estimates would suggest that 5.6% of tuberculosis deaths are caused by TBM. We also estimate that just under half of adults who develop TBM die, a mortality rate far higher than other forms of tuberculosis. Beyond mortality, TBM can place a substantial strain upon individuals, their families, and societies with protracted hospitalisation and half of survivors having long-term neurocognitive disabilities which impair their abilities to work, communicate and function in society [7, 9]. Many require long-term care from either family members, the healthcare system, or social services, at substantial cost to society. Access to rehabilitation is often limited in the populations most affected by tuberculosis. To reduce the burden of mortality, morbidity, and catastrophic costs to families the entire programmatic cascade needs to be evaluated and interventions targeted towards preventing TBM, diagnosing TBM earlier and supporting diagnosed patients with optimal therapy [28]. There also needs to be increased investment in research to identify new vaccines, diagnostics, and therapeutics, including both antimicrobials that effectively penetrate the CSF and host directed therapies to reduce the damage caused by the inflammatory response. A critical next step would be to fully cost the impact of TBM mortality and morbidity as any evaluation of novel diagnostics, therapeutics or vaccination should include the cost of failure to appropriately prevent, diagnose, and treat TBM.

The global epidemiology of adult TBM has commonalities and differences with that of all tuberculosis. HIV infection is a key risk factor for TBM and although only 8.2% of all tuberculosis patients globally are living with HIV, we find that 23% of TBM patients are HIV-positive [29]. Of the 1.4 million individuals who die each year of tuberculosis, 17% were HIV-positive. However, we estimate that 35% of the adults who die of TBM each year were HIV-positive. HIV also impacts the global geography of TBM. While the WHO Africa region contributes 25% of all tuberculosis incidence, it comprises 30% of TBM incidence and 38% of all TBM deaths. The age and sex distribution of TBM in our estimates largely corresponds to the known distribution of all tuberculosis: young adults are at high risk, with consistently higher risk among men in all age groups [30]. The age distribution of TBM deaths is slightly older than TBM incidence reflecting increasing fatality in treated TBM with age.

We carefully considered the strengths and limitations of several strategies to inform the best approach. High quality surveillance data are unavailable for most countries and so it was not possible to use routine data to directly quantify treated TBM incidence or mortality. Many settings endemic for tuberculosis do not use ICD codes to categorise hospital admissions, therefore capturing data on TBM survivors from hospital data was not possible. We were unable to use vital registration data as due to the diverse presentations, difficulties in making a confirmed diagnosis, and scarcity of autopsies and verbal autopsies, a substantial portion of TBM goes unrecognised or is misclassified as another neurological illness. Likewise, there were limited clinical or laboratory data on the TBM fraction in general meningitis cohorts, which tended to focus on either acute epidemic meningitis or HIV-associated meningitis. It was also not possible to use a natural history approach to estimate burden, as has been done for HIV-associated Cryptococcal meningitis, as TBM can present in both people living with HIV (at any CD4 count) and immunocompetent hosts, and also at any age; data to inform natural history parameters in all patient populations is lacking [31]. We therefore chose to use a method that took as its starting point both the estimated and reported tuberculosis incidence for each country, stratified by age, sex, and HIV status and used a meta-analysis of several data sources to estimate the proportion of tuberculosis that is TBM. We assumed the same CDR for TBM as for all tuberculosis and then applied meta-analytic CFRs, calculated using additional data sources and from the literature.

Our study has several limitations. To inform our estimate of the proportion of tuberculosis that is TBM, we obtained data from sources in which data were stratified by age, sex, and HIV status. However, these data sources used different study designs and employed varying inclusion criteria and definitions of TBM. Also, we used these relationships to model the proportion in all countries and it is likely that this relationship varies with multiple factors including tuberculosis prevalence, population demographics, genetics and comorbidities, as well as health system strength. We also assumed that the proportion of tuberculosis that was TBM was the same for diagnosed and undiagnosed tuberculosis. We found no data to inform the magnitude or even direction of the difference between the CDR for TBM and the all-tuberculosis CDR, and so assumed this difference was zero in our primary analysis. We undertook a sensitivity analysis in which we explored the impact of increasing or decreasing the CDR for TBM. Our modelling of CFR in treated TBM was drawn from several studies that stratified case fatality by age, sex and HIV status. The South African and US data were from the routine treatment register and the Vietnam data were from national referral hospital data and clinical trials. We then adjusted these values using data from a systematic review of published studies. None of these data sources are truly representative of the CFR experienced by treated TBM in all global settings. A strength of our work is its independence from vital registration data, which may be of poor quality in some settings.

These estimates suggest that there is a substantial burden of incident TBM and TBM mortality in adults. Over half of adults who die from TBM die undiagnosed, suggesting that to make any form of impact on TBM mortality, increasing access to high-quality diagnostics is required. A substantial proportion of TBM and TBM mortality is HIV-associated so increased HIV testing, improved access to antiretroviral therapy and retention in HIV care would likely reduce TBM risk. We believe that there is a strong rationale for countries to collect routine data on TBM and for more research studies quantifying the long-term neurological deficit in survivors and the cost to societies. There is also a rationale for including an estimate of TBM incidence and mortality in both global estimates of tuberculosis and estimates of meningitis to allow appropriate resource allocation, planning and programme monitoring. Although investment in TBM prevention, diagnostics, and therapeutics are required, many high tuberculosis-burden settings also need improved rehabilitation services. Interventions to reduce the incidence of TBM, and to improve TBM outcomes, are likely to be vital if the ambitious WHO End TB target of reducing tuberculosis deaths by 95% by 2035 are to be met.

## Supporting information

S1 Checklist Guidelines for Accurate and Transparent Health Estimates Reporting (GATHER) checklist. (PDF)

S1 Appendix Appendix containing supplementary methods and results. (PDF)
