# Supplementary material for: The global burden of tuberculous meningitis in adults: A modelling study
Source: PLOS Glob Public Health. 2021 Dec 8;1(12):e0000069. doi: 10.1371/journal.pgph.0000069 (PMC10021871; doi:10.1371/journal.pgph.0000069)
Supplement: S1 Appendix — (PDF) [file pgph.0000069.s002.pdf]

The global burden of tuberculous meningitis in adults: a modelling study (S1 Appendix)

Peter J Dodd, Muhammad Osman, Fiona V Cresswell, Anna M Stadelman, Nguyen Huu Lan, Nguyen Thuy Thuong Thuong, Morris Muzyamba, Lisa Glaser, Sicelo S Dlamini & James A Seddon.

The global burden of tuberculous meningitis in adults: a modelling study (Appendix) 1

Supplementary details of methods 1

Online sources 1

List of countries included 1

Supplementary results 2

Supplementary tables 2

Table 1 Meta-analysis estimated percentages (Figure 2 data) with standard deviation in brackets. 2

Table 2. Sensitivity analysis results with odds ratio of 2 for TBM detection & treatment relative to all TB (hn=HIV-ve; hp=HIV+ve) 5

Table 3. Sensitivity analysis results with odds ratio of 0.5 for TBM detection & treatment relative to all TB (hn=HIV-ve; hp=HIV+ve) 7

Supplementary details of methods

Online sources

WHO TB estimates and data can be downloaded from https://www.who.int/teams/global-tuberculosis-programme/data
(analysis versions accessed 24/02/2020 are included in the repository indata/ folder).

United Nations World Population Prospects 2019 data was accessed from https://population.un.org/wpp/Download/Standard/Population/
(a version in R Rdata format is included in the repository indata/ folder).

List of countries included

ISO3 codes for the 152 included countries:

AFG, ALB, ARG, ARM, AUS, AUT, AZE, BDI, BEL, BEN, BFA, BGD, BGR, BIH, BLR, BOL, BRA, BRN, BTN, BWA, CAF, CAN, CHE, CHL, CHN, CIV, CMR, COD, COG, COL, CRI, CUB, CZE, DEU, DJI, DNK, DOM, DZA, ECU, EGY, ERI, ESP, ETH, FIN, FJI, FRA, GAB, GBR, GEO, GHA, GIN, GNB, GNQ, GRC, GTM, GUY, HKG, HND, HRV, HTI, HUN, IDN, IND, IRL, IRN, IRQ, ISR, ITA, JOR, JPN, KAZ, KEN, KGZ, KHM, KIR, KOR, KWT, LAO, LBN, LBR, LBY, LKA, LSO, LTU, MAC, MAR, MDA, MDG, MEX, MHL, MKD, MLI, MMR, MNG, MRT, MWI, MYS, NAM, NER, NGA, NIC, NLD, NPL, NZL, OMN, PAK, PAN, PER, PHL, PNG, POL, PRK, PRT, PRY, QAT, ROU, RUS, RWA, SAU, SDN, SGP, SLB, SLE, SLV, SOM, SRB, SSD, SVK, SWE, SWZ, SYR, TCD, TGO, THA, TJK, TKM, TLS, TTO, TUN, TUR, TZA, UGA, UKR, URY, USA, UZB, VEN, VNM, YEM, ZAF, ZMB, ZWE

Supplementary results

Supplementary tables

Table 1 Meta-analysis estimated percentages (Figure 2 data) with standard deviation in brackets.

NB Brazil data for both sexes combined

	hiv	sex	age	pooled	BRA	GBR	USA	VNM	ZAF
**cfr**	**hiv+**	**M**	**15-24**	53.1 (5.1)				58.1 (5)	47.4 (5.3)
**cfr**	**hiv+**	**M**	**25-34**	52.7 (3)				51.4 (2.7)	57.9 (5.6)
**cfr**	**hiv+**	**M**	**35-44**	49.8 (4.4)				44.1 (4)	62.5 (6.1)
**cfr**	**hiv+**	**M**	**45-54**	67.1 (7.8)				58.1 (9.3)	72.5 (7.2)
**cfr**	**hiv+**	**M**	**55-64**	97.4 (10.1)				0 (24.9)	97.4 (10.1)
**cfr**	**hiv+**	**M**	**65+**	100 (13.3)					100 (13.3)
**cfr**	**hiv+**	**F**	**15-24**	40.2 (4.5)				53.3 (13.3)	38.8 (4.3)
**cfr**	**hiv+**	**F**	**25-34**	50.1 (5.1)				54.7 (6.5)	47.7 (4.7)
**cfr**	**hiv+**	**F**	**35-44**	52.1 (5.5)				54.2 (10.6)	51.6 (5.1)
**cfr**	**hiv+**	**F**	**45-54**	52.2 (6.5)				12.5 (13.4)	60.3 (6)
**cfr**	**hiv+**	**F**	**55-64**	82.2 (8.7)					82.2 (8.7)
**cfr**	**hiv+**	**F**	**65**	100 (13.9)					100 (13.9)
**cfr**	**hiv-**	**M**	**15-24**	7.8 (1.2)			14.2 (4.9)	16.2 (4.6)	4.7 (0.7)

**cfr**	**hiv-**	**M**	**25-34**	11.5 (1.2)			20.2 (4)	13.3 (3.5)	7.1 (0.9)
**cfr**	**hiv-**	**M**	**35-44**	14.8 (1.2)			21.7 (3.9)	21.8 (3.9)	11.1 (1.4)
**cfr**	**hiv-**	**M**	**45-54**	19.7 (1.3)			30.3 (4.6)	31.6 (5.5)	15.1 (1.6)
**cfr**	**hiv-**	**M**	**55-64**	23.1 (1.3)			31.9 (4.9)	29.2 (5.6)	19.5 (2.1)
**cfr**	**hiv-**	**M**	**65+**	35.1 (1.4)			47.9 (5.1)	37.5 (6.3)	29.6 (3)
**cfr**	**hiv-**	**F**	**15-24**	6.7 (1.2)			14.4 (4.9)	7.1 (3.1)	4.1 (0.6)
**cfr**	**hiv-**	**F**	**25-34**	10.4 (1.2)			20.4 (4.6)	23.5 (5)	6.3 (0.8)
**cfr**	**hiv-**	**F**	**35-44**	13.7 (1.2)			21.9 (4.2)	22.6 (5.6)	9.8 (1.2)
**cfr**	**hiv-**	**F**	**45-54**	18.6 (1.3)			30.5 (5)	25.9 (6.3)	13.4 (1.6)
**cfr**	**hiv-**	**F**	**55-64**	21.9 (1.3)			32.1 (5)	26.3 (7.6)	17.4 (2)
**cfr**	**hiv-**	**F**	**65+**	34 (1.4)			48.2 (5)	43.3 (6.3)	26.8 (3)
**prop**	**hiv+**	**M**	**15-24**	5 (2.4)				9.8 (2.5)	1.7 (0.1)
**prop**	**hiv+**	**M**	**25-34**	5.6 (2.4)				10.1 (1.9)	1.7 (0)
**prop**	**hiv+**	**M**	**35-44**	5.4 (2.4)				9.4 (1.7)	1.7 (0)
**prop**	**hiv+**	**M**	**45-54**	4.5 (2.4)				7.6 (1.8)	1.6 (0)
**prop**	**hiv+**	**M**	**55-64**	4.6 (2.5)				9 (2.8)	1.5 (0.1)
**prop**	**hiv+**	**M**	**65+**	3.6 (2.6)				8.3 (4.4)	1.2 (0.1)
**prop**	**hiv+**	**F**	**15-24**	6.2 (2.3)	4.1 (0.4)			16 (3.9)	2.2 (0.1)
**prop**	**hiv+**	**F**	**25-34**	6.8 (2.3)	4.6 (0.3)			16.5 (3)	2.2 (0.1)
**prop**	**hiv+**	**F**	**35-44**	6.6 (2.2)	4.9 (0.4)			15.4 (2.9)	2.2 (0.1)
**prop**	**hiv+**	**F**	**45-54**	5.8 (2.3)	5.1 (0.4)			12.6 (3.1)	2.1 (0.1)

**prop**	**hiv+**	**F**	**55-64**	5.8 (2.3)	5 (0.4)			14.8 (4.5)	1.9 (0.1)
**prop**	**hiv+**	**F**	**65+**	4.8 (2.4)	5.4 (0.5)			13.6 (6.7)	1.5 (0.1)
**prop**	**hiv-**	**M**	**15-24**	1.5 (0.3)		2 (0.1)	1.4 (0.2)	1.8 (0.2)	0.7 (0)
**prop**	**hiv-**	**M**	**25-34**	1.4 (0.3)		1.8 (0.1)	1.5 (0.2)	1.5 (0.2)	0.7 (0)
**prop**	**hiv-**	**M**	**35-44**	1.7 (0.3)		2.2 (0.1)	2 (0.2)	2 (0.3)	0.6 (0)
**prop**	**hiv-**	**M**	**45-54**	1.5 (0.3)		2.1 (0.1)	1.7 (0.2)	1.9 (0.2)	0.5 (0)
**prop**	**hiv-**	**M**	**55-64**	1.6 (0.3)		2.4 (0.1)	1.4 (0.2)	2.1 (0.3)	0.5 (0)
**prop**	**hiv-**	**M**	**65+**	1.7 (0.4)		1.9 (0.1)	1.1 (0.1)	4 (0.5)	0.4 (0)
**prop**	**hiv-**	**F**	**15-24**	1.6 (0.3)	0.5 (0.1)	2.2 (0.1)	1.7 (0.2)	2.7 (0.4)	1 (0.1)
**prop**	**hiv-**	**F**	**25-34**	1.5 (0.3)	0.5 (0.1)	2.1 (0.1)	1.8 (0.2)	2.2 (0.3)	1 (0.1)
**prop**	**hiv-**	**F**	**35-44**	1.8 (0.3)	0.5 (0.1)	2.5 (0.1)	2.4 (0.3)	3 (0.4)	0.8 (0.1)
**prop**	**hiv-**	**F**	**45-54**	1.7 (0.3)	0.6 (0.1)	2.4 (0.1)	2 (0.2)	2.7 (0.3)	0.7 (0.1)
**prop**	**hiv-**	**F**	**55-64**	1.7 (0.3)	0.7 (0.1)	2.7 (0.2)	1.8 (0.2)	3.1 (0.4)	0.7 (0.1)
**prop**	**hiv-**	**F**	**65+**	1.8 (0.3)	0.7 (0.1)	2.2 (0.1)	1.3 (0.1)	5.8 (0.7)	0.5 (0)

Table 2. Sensitivity analysis results with odds ratio of 2 for TBM detection & treatment relative to all TB (hn=HIV-ve; hp=HIV+ve)

WHO region	AFR	AMR	EMR	EUR	SEA	WPR	Global
**hn.treated**	12,000 (11,500 - 12,500)	3,190 (3,010 - 3,370)	7,110 (6,490 - 7,740)	3,030 (2,870 - 3,200)	45,000 (41,100 - 49,000)	21,200 (19,300 - 23,000)	91,600 (87,100 - 96,000)
**hp.treated**	16,800 (11,900 - 21,700)	1,180 (0 - 2,570)	209 (0 - 4,550)	1,380 (0 - 2,790)	5,020 (0 - 33,300)	1,470 (0 - 14,600)	26,000 (0 - 58,000)
**treated**	28,800 (23,900 - 33,700)	4,370 (2,970 - 5,760)	7,320 (2,930 - 11,700)	4,420 (3,000 - 5,830)	50,100 (21,500 - 78,700)	22,600 (9,420 - 35,900)	118,000 (85,400 - 150,000)
**hn.untreated**	4,970 (3,620 - 6,320)	333 (187 - 479)	2,040 (737 - 3,340)	218 (34 - 402)	7,170 (887 - 13,400)	2,660 (566 - 4,750)	17,400 (10,500 - 24,300)
**hp.untreated**	4,690 (2,860 - 6,510)	120 (63 - 177)	56 (24 - 88)	74 (0 - 226)	733 (0 - 1,510)	205 (70 - 340)	5,880 (3,880 - 7,870)
**untreated**	9,660 (7,390 - 11,900)	454 (297 - 610)	2,100 (792 - 3,400)	292 (54 - 531)	7,900 (1,570 - 14,200)	2,860 (767 - 4,960)	23,300 (16,100 - 30,400)
**hn.total**	17,000 (15,600 - 18,400)	3,520 (3,290 - 3,750)	9,150 (7,710 - 10,600)	3,250 (3,000 - 3,500)	52,200 (44,800 - 59,600)	23,800 (21,100 - 26,600)	109,000 (101,000 - 117,000)
**hp.total**	21,500 (16,300 - 26,700)	1,300 (0 - 2,690)	265 (0 - 4,610)	1,460 (45 - 2,870)	5,750 (0 - 34,100)	1,680 (0 - 14,800)	31,900 (0 - 63,900)
**total**	38,500 (33,100 - 43,900)	4,820 (3,410 - 6,230)	9,420 (4,840 - 14,000)	4,710 (3,280 - 6,140)	58,000 (28,700 - 87,300)	25,500 (12,100 - 38,900)	141,000 (108,000 - 174,000)
**deaths.hn.treated**	1,880 (1,800 - 1,960)	543 (512 - 575)	1,190 (1,080 - 1,300)	545 (516 - 575)	7,680 (6,970 - 8,380)	4,350 (3,880 - 4,810)	16,200 (15,300 - 17,000)
**deaths.hp.treated**	9,700 (6,890 - 12,500)	720 (0 - 1,590)	127 (0 - 2,920)	846 (0 - 1,700)	3,090 (0 - 21,300)	997 (0 - 11,800)	15,500 (0 - 37,100)
**deaths.treated**	11,600 (8,770 - 14,400)	1,260 (391 - 2,140)	1,320 (0 - 4,110)	1,390 (533 - 2,250)	10,800 (0 - 29,000)	5,340 (0 - 16,100)	31,700 (10,100 - 53,300)
**deaths.hn.untreated**	4,970 (3,620 - 6,320)	333 (187 - 479)	2,040 (737 - 3,340)	218 (34 - 402)	7,170 (887 - 13,400)	2,660 (566 - 4,750)	17,400 (10,500 - 24,300)
**deaths.hp.untreated**	4,690 (2,860 - 6,510)	120 (63 - 177)	56 (24 - 88)	74 (0 - 226)	733 (0 - 1,510)	205 (70 - 340)	5,880 (3,880 - 7,870)
**deaths.untreated**	9,660 (7,390 - 11,900)	454 (297 - 610)	2,100 (792 - 3,400)	292 (54 - 531)	7,900 (1,570 - 14,200)	2,860 (767 - 4,960)	23,300 (16,100 - 30,400)
**deaths.hn.total**	6,850 (5,490 - 8,210)	877 (728 - 1,030)	3,230 (1,920 - 4,540)	763 (577 - 950)	14,800 (8,520 - 21,200)	7,000 (4,860 - 9,140)	33,600 (26,600 - 40,500)
**deaths.hp.total**	14,400 (11,000 - 17,700)	840 (0 - 1,710)	183 (0 - 2,970)	920 (49 - 1,790)	3,820 (0 - 22,100)	1,200 (0 - 12,000)	21,400 (0 - 43,000)

**deaths.total**	21,200 (17,600 - 24,900)	1,720 (830 - 2,600)	3,410 (334 - 6,490)	1,680 (792 - 2,580)	18,700 (0 - 38,000)	8,200 (0 - 19,200)	54,900 (32,200 - 77,700)

Table 3. Sensitivity analysis results with odds ratio of 0.5 for TBM detection & treatment relative to all TB (hn=HIV-ve; hp=HIV+ve)

WHO region	AFR	AMR	EMR	EUR	SEA	WPR	Global
**hn.treated**	12,000 (11,500 - 12,500)	3,190 (3,010 - 3,370)	7,110 (6,490 - 7,740)	3,030 (2,870 - 3,200)	45,000 (41,100 - 49,000)	21,200 (19,300 - 23,000)	91,600 (87,100 - 96,000)
**hp.treated**	16,800 (11,900 - 21,700)	1,180 (0 - 2,570)	209 (0 - 4,550)	1,380 (0 - 2,790)	5,020 (0 - 33,300)	1,470 (0 - 14,600)	26,000 (0 - 58,000)
**treated**	28,800 (23,900 - 33,700)	4,370 (2,970 - 5,760)	7,320 (2,930 - 11,700)	4,420 (3,000 - 5,830)	50,100 (21,500 - 78,700)	22,600 (9,420 - 35,900)	118,000 (85,400 - 150,000)
**hn.untreated**	19,900 (14,500 - 25,300)	1,330 (750 - 1,920)	8,160 (2,950 - 13,400)	872 (136 - 1,610)	28,700 (3,550 - 53,800)	10,600 (2,260 - 19,000)	69,500 (42,000 - 97,100)
**hp.untreated**	18,700 (11,400 - 26,000)	481 (253 - 710)	223 (97 - 350)	297 (0 - 905)	2,930 (0 - 6,040)	821 (281 - 1,360)	23,500 (15,500 - 31,500)
**untreated**	38,600 (29,500 - 47,700)	1,810 (1,190 - 2,440)	8,380 (3,170 - 13,600)	1,170 (214 - 2,120)	31,600 (6,290 - 56,900)	11,400 (3,070 - 19,800)	93,000 (64,400 - 122,000)
**hn.total**	31,900 (26,500 - 37,300)	4,520 (3,910 - 5,130)	15,300 (10,000 - 20,500)	3,900 (3,150 - 4,660)	73,700 (48,300 - 99,100)	31,800 (23,200 - 40,400)	161,000 (133,000 - 189,000)
**hp.total**	35,500 (26,800 - 44,300)	1,660 (255 - 3,070)	432 (0 - 4,780)	1,680 (150 - 3,210)	7,950 (0 - 36,400)	2,290 (0 - 15,400)	49,600 (16,600 - 82,500)
**total**	67,400 (57,100 - 77,800)	6,180 (4,650 - 7,710)	15,700 (8,890 - 22,500)	5,590 (3,880 - 7,290)	81,700 (43,500 - 120,000)	34,100 (18,400 - 49,700)	211,000 (168,000 - 254,000)
**deaths.hn.treated**	1,880 (1,800 - 1,960)	543 (512 - 575)	1,190 (1,080 - 1,300)	545 (516 - 575)	7,680 (6,970 - 8,380)	4,350 (3,880 - 4,810)	16,200 (15,300 - 17,000)
**deaths.hp.treated**	9,700 (6,890 - 12,500)	720 (0 - 1,590)	127 (0 - 2,920)	846 (0 - 1,700)	3,090 (0 - 21,300)	997 (0 - 11,800)	15,500 (0 - 37,100)
**deaths.treated**	11,600 (8,770 - 14,400)	1,260 (391 - 2,140)	1,320 (0 - 4,110)	1,390 (533 - 2,250)	10,800 (0 - 29,000)	5,340 (0 - 16,100)	31,700 (10,100 - 53,300)
**deaths.hn.untreated**	19,900 (14,500 - 25,300)	1,330 (750 - 1,920)	8,160 (2,950 - 13,400)	872 (136 - 1,610)	28,700 (3,550 - 53,800)	10,600 (2,260 - 19,000)	69,500 (42,000 - 97,100)
**deaths.hp.untreated**	18,700 (11,400 - 26,000)	481 (253 - 710)	223 (97 - 350)	297 (0 - 905)	2,930 (0 - 6,040)	821 (281 - 1,360)	23,500 (15,500 - 31,500)
**deaths.untreated**	38,600 (29,500 - 47,700)	1,810 (1,190 - 2,440)	8,380 (3,170 - 13,600)	1,170 (214 - 2,120)	31,600 (6,290 - 56,900)	11,400 (3,070 - 19,800)	93,000 (64,400 - 122,000)
**deaths.hn.total**	21,800 (16,300 - 27,200)	1,880 (1,290 - 2,460)	9,350 (4,140 - 14,600)	1,420 (681 - 2,150)	36,300 (11,200 - 61,500)	15,000 (6,600 - 23,300)	85,700 (58,200 - 113,000)

**deaths.hp.total**	28,400 (20,600 - 36,300)	1,200 (300 - 2,100)	350 (0 - 3,140)	1,140 (91 - 2,200)	6,020 (0 - 24,500)	1,820 (0 - 12,600)	39,000 (16,000 - 62,000)
**deaths.total**	50,200 (40,700 - 59,700)	3,080 (2,000 - 4,150)	9,700 (3,790 - 15,600)	2,560 (1,280 - 3,840)	42,400 (11,200 - 73,600)	16,800 (3,140 - 30,400)	125,000 (88,800 - 161,000)
